# The Prophylactic Protection of *Salmonella* Typhimurium Infection by *Lentilactobacillus buchneri* GX0328-6 in Mice

**DOI:** 10.1007/s12602-023-10145-8

**Published:** 2023-09-05

**Authors:** Yan Shi, Hao Peng, Yuying Liao, Jun Li, Yangyan Yin, Hongyan Peng, Leping Wang, Yizhou Tan, Changting Li, Huili Bai, Chunxia Ma, Wenbao Tan, Xun Li

**Affiliations:** 1 College of Animal Science and Technology, Guangxi University, Nanning, 530004 China; 2 Guangxi Key Laboratory of Veterinary Biotechnology, Guangxi Veterinary Research Institute, Nanning, 530001 China; 3 Fangchenggang Administrative Examination and Approval Service Center, Fangchenggang, 538001 Guangxi China; 4 Key Laboratory of China (Guangxi)-ASEAN Cross-Border Animal Disease Prevention and Control, Ministry of Agriculture and Rural Affairs of China, Nanning, 530021 China; 5 Qibainong Chicken Industry Development Center of Dahua Yao Autonomous County, Dahua Guangxi, 530800 China

**Keywords:** *Lentilactobacillus buchneri*, *Salmonella* typhimurium, Gut microbiota, Gene expression

## Abstract

Salmonellosis is a disease caused by non-typhoid Salmonella, and although some lactic acid bacteria strains have been shown previously to relieve Salmonellosis symptoms, little has been studied about the preventive mechanism of *Lentilactobacillus buchneri* (*L. buchneri*) against *Salmonella* infection in vivo. Therefore, the *L. buchneri* was fed to C57BL/6 mice for 10 days to build a protective system of mice to study its prevention and possible mechanisms. The results showed that *L. buchneri* GX0328-6 alleviated symptoms caused by *Salmonella* typhimurium infection among C57BL/6 mice, including low survival rate, weight loss, increase in immune organ index and hepatosplenomegaly, and modulated serum immunoglobulin levels and intrinsic immunity. Importantly, the *L. buchneri* GX0328-6 enhanced the mucosal barrier of the mouse jejunum by upregulating the expression of tight junction proteins such as ZO-1, occludins, and claudins-4 and improved absorptive capacity by increasing the length of mouse jejunal villus and the ratio of villus length to crypt depth and decreasing the crypt depth. *L. buchneri* GX0328-6 reduced the intestinal proliferation and invasion of *Salmonella* typhimurium by modulating the expression of antimicrobial peptides in the intestinal tract of mice, and reduced intestinal inflammation and systemic spread in mice by downregulating the expression of IL-6 and promoting the expression of IL-10. Furthermore, *L. buchneri* GX0328-6 increased the relative abundance of beneficial bacteria colonies and decreased the relative abundance of harmful bacteria in the cecum microflora by modulating the microflora in the cecum contents.

**Supplementary Information:**

The online version contains supplementary material available at 10.1007/s12602-023-10145-8.

## Introduction

In the last two decades, *Salmonella* was considered the main pathogen of foodborne diseases worldwide including gastroenteritis [1, 2]. *Salmonella* stimulates the expression of immune system genes, which involve in the inflammatory responses [3, 4]. *Salmonella enterica* serovar typhimurium causes non-typhoidal salmonellosis, which is one of the most prevalent foodborne diseases [5]. As an important intracellular bacterium, the infection of *Salmonella* typhimurium is not only a serious threat to human health but also causes significant economic losses in the agriculture industry [6, 7]. Although salmonellosis can be treated with multiple types of broad-spectrum antibiotics, the limitation includes antibiotic resistance and food safety issues [8–11]. Therefore, there is an urgent need to find alternative strategies to reduce the transmission of foodborne pathogens in a safer and eco-friendly way [12].

Probiotics have been broadly used in producing food such as cheese, yogurt, and fermented salted fish [13–16]. The health benefits of fermented food have been confirmed. Probiotics have been described as “organisms and substances that contribute to intestinal microbial homeostasis” [17, 18]. It modulates the immune system, limits pathogen colonization, and controls inflammatory bowel diseases and metabolic disorders [19]. Previous studies found that probiotics alter the mucosal immune system through Toll-like receptor-mediated processes. They promote T helper cell 1 differentiation thereby increasing antibody production, inducing phagocytosis, and enhancing the activities of natural cells. Probiotics can also inhibit nuclear factor light chain enhancers to activate B cell pathways, induce T cell apoptosis, increase anti-inflammatory cytokines such as interleukin (IL)-10, and decrease pro-inflammatory cytokines [19–24]. Previous studies also showed that *Lactobacillus plantarum* P8 could inhibit oocyst shedding as well as improve the general growth performance and intestinal health among broilers infected by *Eimeria coccidia* [25]. Wang et al. showed that *Lactobacillus plantarum* could attenuate *Clostridium perfringens*–induced growth performances and soothe intestinal ecological disorders among broilers by an increased level of short-chain fatty acids therefore improving their intestinal health [26]. Previous studies also showed that taking dietary with *Lactobacillus plantarum* B1 could increase the number of lactic acid bacteria and the concentration of short-chain fatty acid (SCFA) concentrations in the intestine [27]. Mazkour et al. found that the supplementation with both *Bacillus subtilis* and *Bacillus coagulans* improved the growth performance through benefiting intestinal microorganisms in rats [28]. Jang et al. found that the *Lactobacillus fermentum* improved mice’s immune system by modulating their intestinal flora [29]. Wang et al. represented that *Lactobacillus reuteri* promoted intestinal development and modulated mucosal immunity in neonatal piglets [30]. In general, different probiotic strains can either promote the growth performance of animals or attenuate the tissue damage caused by foreign pathogens and soothe their intestinal ecological dysregulations.

The *Lentilactobacillus buchneri* could metabolize lactic acid into acetic acid and 1,2-propanediol under the anaerobic and acidic conditions, therefore maintaining cell activities. In the storage of sugarcane silage, the *L. buchneri* reduces the loss of dry matter content, maintains its pH value, benefits the lactic acid bacteria (LAB) population, helps the dry matter recovery, and maintains the concentration of WSC, lactic acid, acetic acid, and ethanol concentrations within a healthy range [31–34]. In general, the application of *L. buchneri* has strong advantages in feed preservation, particularly in reducing dry matter loss, improving aerobic stability and degradation rate [35, 36]. Additionally, adding *L. buchneri* also has a lower cost compared to chemical additives [37, 38]. It has also been reported that *L. buchneri* promotes the growth performance of cattle and regulates the microbial population of silage [31, 35]. However, little is known about its regulation of intestinal mucosal barriers such as microflora in monogastric animals, and neither does the mechanism of *L. buchneri* in the prevention of *Salmonella* typhimurium infection, nor its probiotic potential has been well studied. Therefore, this study tends to investigate the preventive and protective effects of *L. buchneri* in improving the function of intestinal mucosal barriers and its immune functions against *Salmonella* typhimurium infection among C57/BL6 mice.

## Results

### Effects of L. buchneri GX0328-6 on Immune Organs, Body Weights, and Survival Rates

**Fig. 1:**
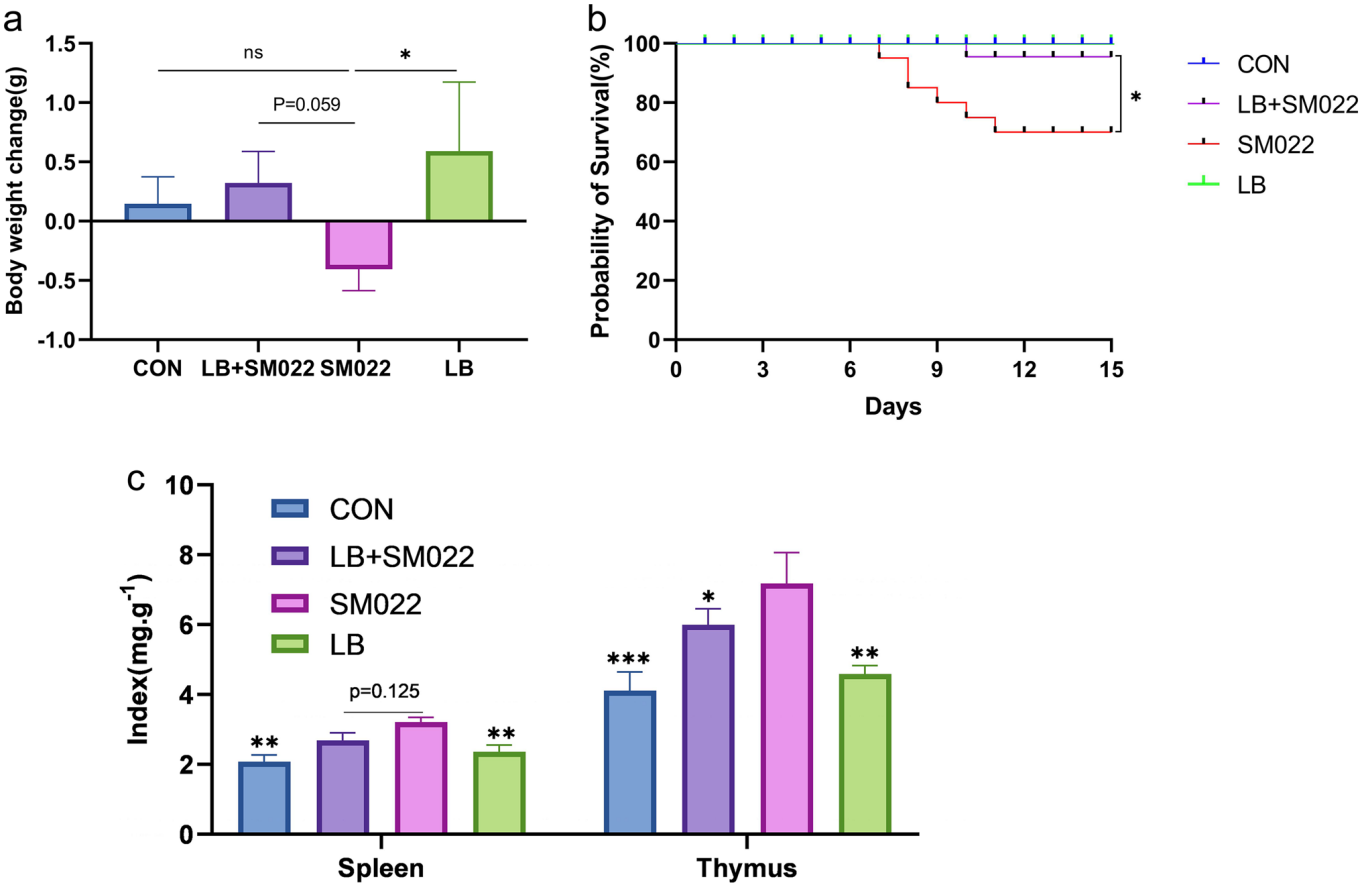
The body weight change (**a**), survival rate (**b**), and immune organ index (**c**) in mice treated or not during 10 days by *L. buchneri* GX0328-6 and then infected or not with *Salmonella* typhimurium SM022. The negative control group (CON) and *L. buchneri* group (LB) were only fed with saline and *L. buchneri* GX0328-6 respectively for 10 consecutive days; the prophylactic group (LB + SM022) and the positive group (SM022) were given *L. buchneri* GX0328-6 and saline respectively for 10 consecutive days, and then the mice were fed with *Salmonella* typhimurium SM022. *P < 0.05, ns had no statistical significance. (one-way ANOVA)

Figure 1a shows that oral administration of *L. buchneri* GX0328-6 affects body weight changes in *Salmonella* typhimurium–infected mice. On day 5 after infection, body weight significantly decreased among mice in the positive group (SM022) (*P* < 0.05). Pretreatment with *L. buchneri* GX0328-6 reduced the weight loss caused by the infection of *Salmonella* typhimurium (*P* = 0.059). Figure 1b shows that the oral administration of *L. buchneri* GX0328-6 could improve the survival rate of *Salmonella* typhimurium–infected mice. The survival rate was 100% in both the negative control (CON) and *L. buchneri*-treated groups, 91.67% in the prophylactic group (LB + SM022) while only 50% of the positive group infected with *Salmonella* typhimurium survived. In addition, *L. buchneri* GX0328-6 pretreatment reduced the thymic and splenic indices in mice, especially the thymic index (*P* < 0.05) (Fig. 1c).

### The probiotic L. buchneri GX0328-6 Reduces Transmission of Salmonella Typhimurium Among C57BL/6 Mice

**Fig. 2:**
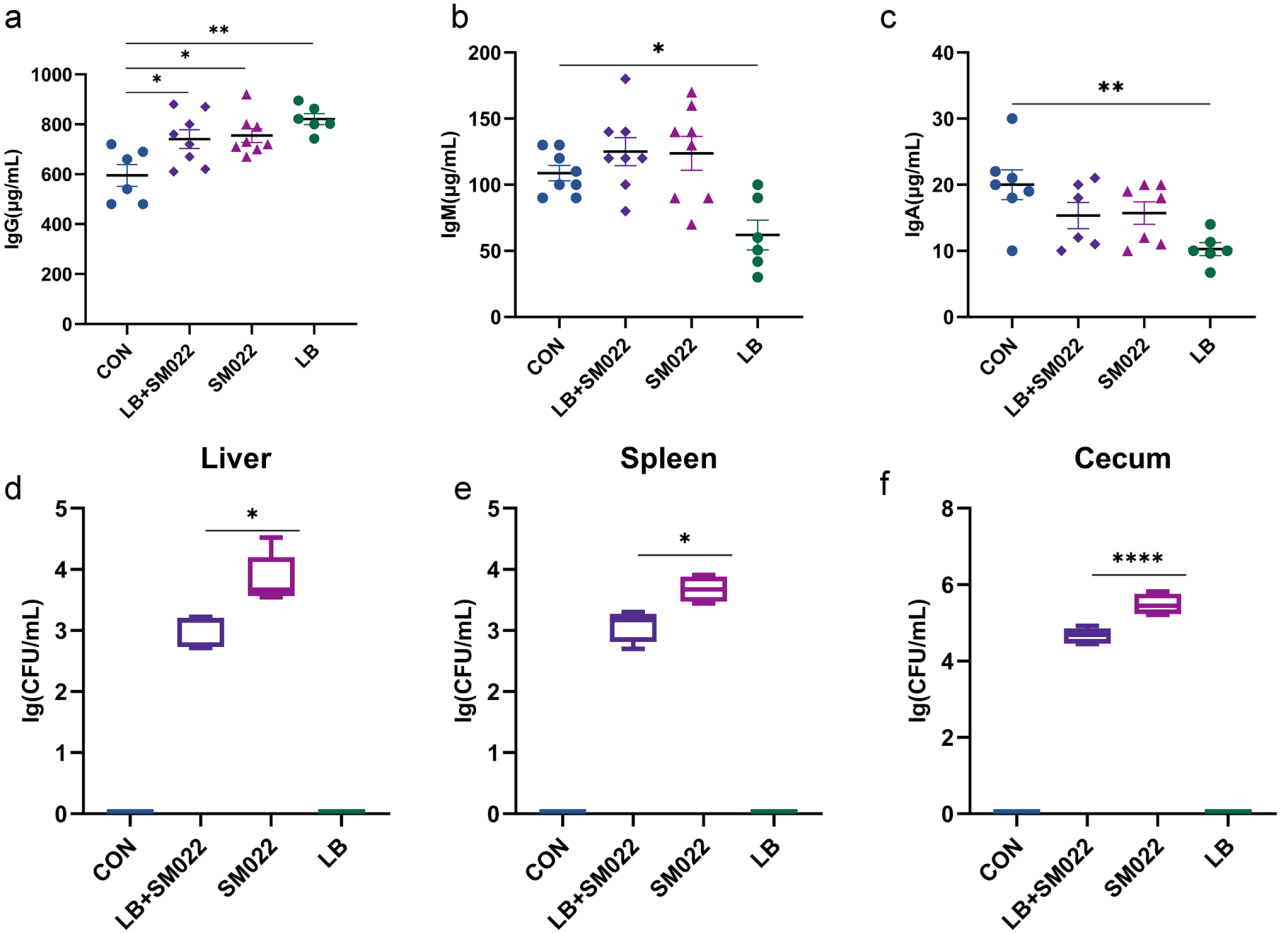
The levels of immunoglobulin IgG (**a**), IgM (**b**), and IgA (**c**) in mice treated or not during 10 days by *L. buchneri* GX0328-6 and then infected or not with *Salmonella* typhimurium SM022, the contents of *Salmonella* typhimurium in mouse liver (**d**), spleen (**e**) and cecum (**f**) tissues in mice treated or not during 10 days by *L. buchneri* GX0328-6 and then infected with *Salmonella* typhimurium SM022 (*n* = 10) *P < 0.05, **P < 0.01, ****P < 0.0001, ns had no statistical significance (one-way ANOVA)

Compared to the negative control group, we found that the IgG level was significantly increased (*P* < 0.05) among *Salmonella* typhimurium–infected mice. Meanwhile, the levels of IgG, IgA, and IgM in mice infected with *Salmonella* typhimurium after pretreatment with *L. buchneri* GX0328-6 had no significant difference compared with the positive group. However, when treated with *L. buchneri* GX0328-6 only, mice showed a significant increase in IgG (*P* < 0.01) while the level of both IgA and IgM were significantly decreased (*P* < 0.05) (Fig. 2a, b, and c). Interestingly, among *L. buchneri* GX0328-6 pretreated mice, we found that pathogen translocation was significantly reduced in the spleen, the liver, and the cecum compared to the positive group; particularly, the largest amount of reduction was observed in their cecum (Fig. 2d, e, f).

### Pretreating with L. buchneri GX0328-6 Could Reduce the Severity of Salmonella Typhimurium Infection Among C57BL/6 Mice

**Fig. 3:**
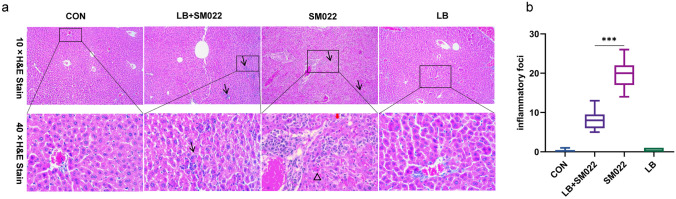
The representative liver micrograph of mice treated or not during 10 days by *L. buchneri* GX0328-6 and then infected with *Salmonella* typhimurium SM022. The black thin arrow indicates that there are inflammatory cells (10 ×); the red thick arrow indicates necrosis of liver cells (40 ×); the black triangle box indicates the aggregation of red blood cells (40 ×). b is the result of liver inflammation–related cell frequency. ***P < 0.001 (one-way ANOVA)

**Fig. 4:**
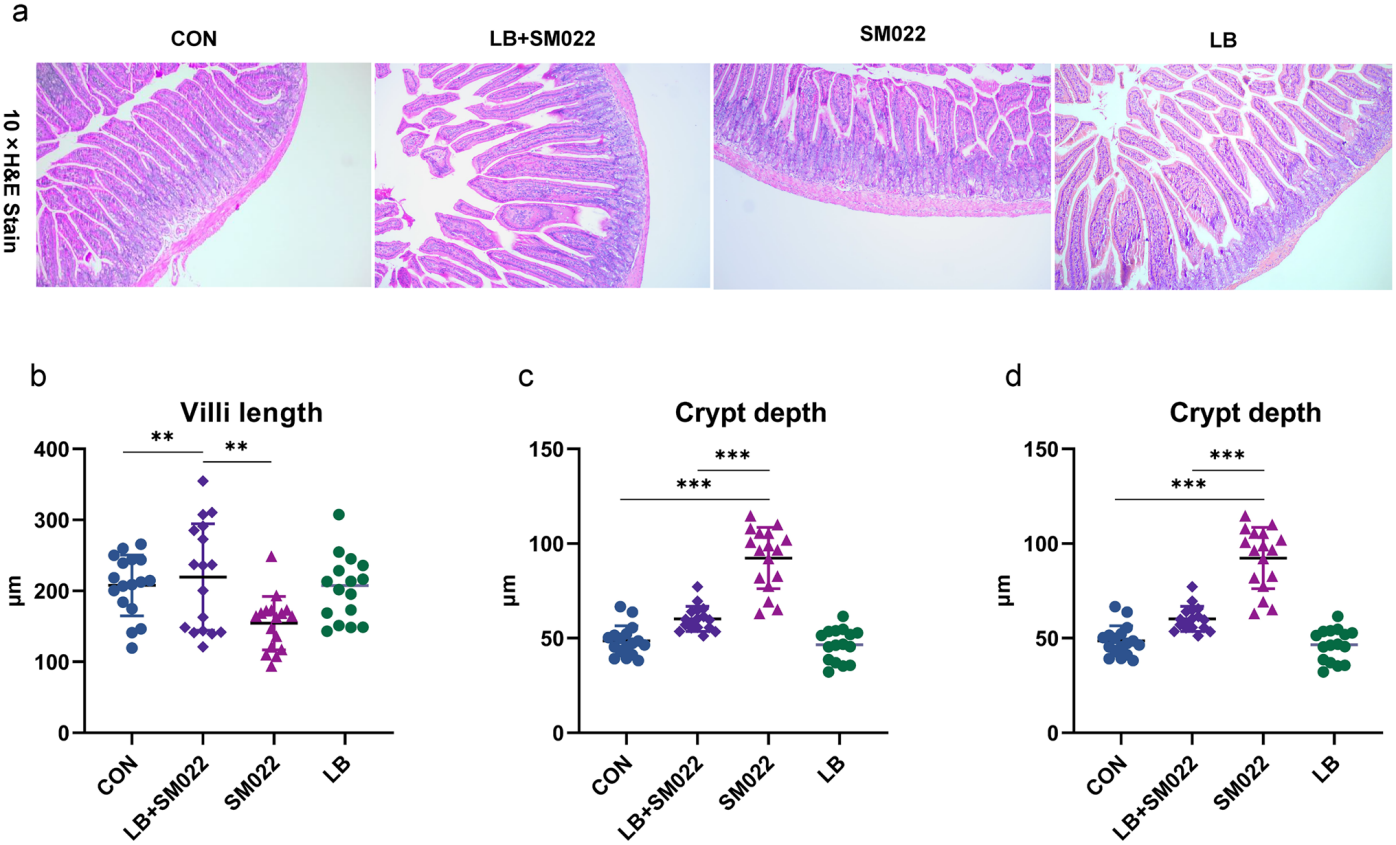
The pathological changes (**a**) of jejunum mucosa in mice, the villus length (**b**), the crypt depth (**c**) and the ratio (**d**) of villus and crypt depth in the jejunum of mice treated or not during 10 days by *L. buchneri* GX0328-6 and then infected with *Salmonella* typhimurium SM022. **P < 0.01; ***P < 0.001 (one-way ANOVA)

Compared with the prophylactic group, the liver and spleen of positive controls were significantly enlarged, and the color of these livers became lighter (Appendix 1a). In addition, reduced food intake occurs among mice in the positive group with reduced movements and self-grooming, as well as disheveled hair that lost its luster (Appendix 1b). We also investigated the morphological changes of the liver under infection by evaluating the frequency and severity of inflammatory foci. We observed the decrease of inflammatory cell number and hepatocyte necrosis in the liver of mice in the prophylactic group compared to the positive group (Fig. 3). In addition, small intestinal villi and the ratio of small intestinal villi to crypt depth were significantly increased, and crypt depth was significantly decreased in the prophylactic group compared to the positive group (Fig. 4).

### L. buchneri GX0328-6 Regulates mRNA Expression of Inflammatory Cytokines, Antimicrobial Peptides, and Intestinal Mucosa–Associated Proteins During Salmonella Typhimurium C57BL/6 Infection

**Fig. 5:**
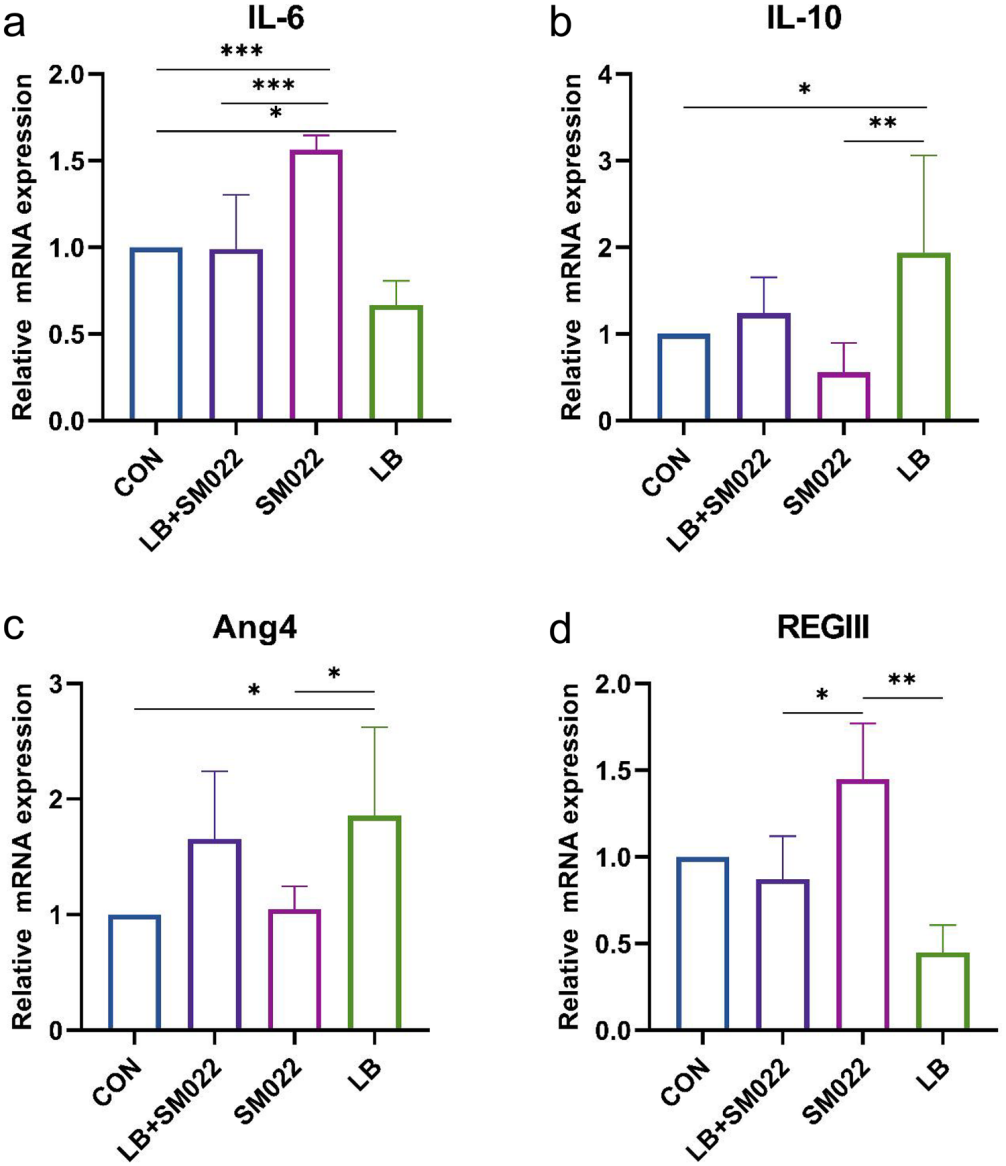
mRNA relative expression amounts of inflammation-related cytokines IL-6 (a) and IL-10 (b) and antibacterial peptides Ang4 (c) and REGIII (d) in the jejunum of mice treated or not during 10 days by *L. buchneri* GX0328-6 and then infected or not with *Salmonella* typhimurium SM022. (*n* = 6) *P < 0.05, **P < 0.01, ***P < 0.001 (one-way ANOVA)

**Fig. 6:**
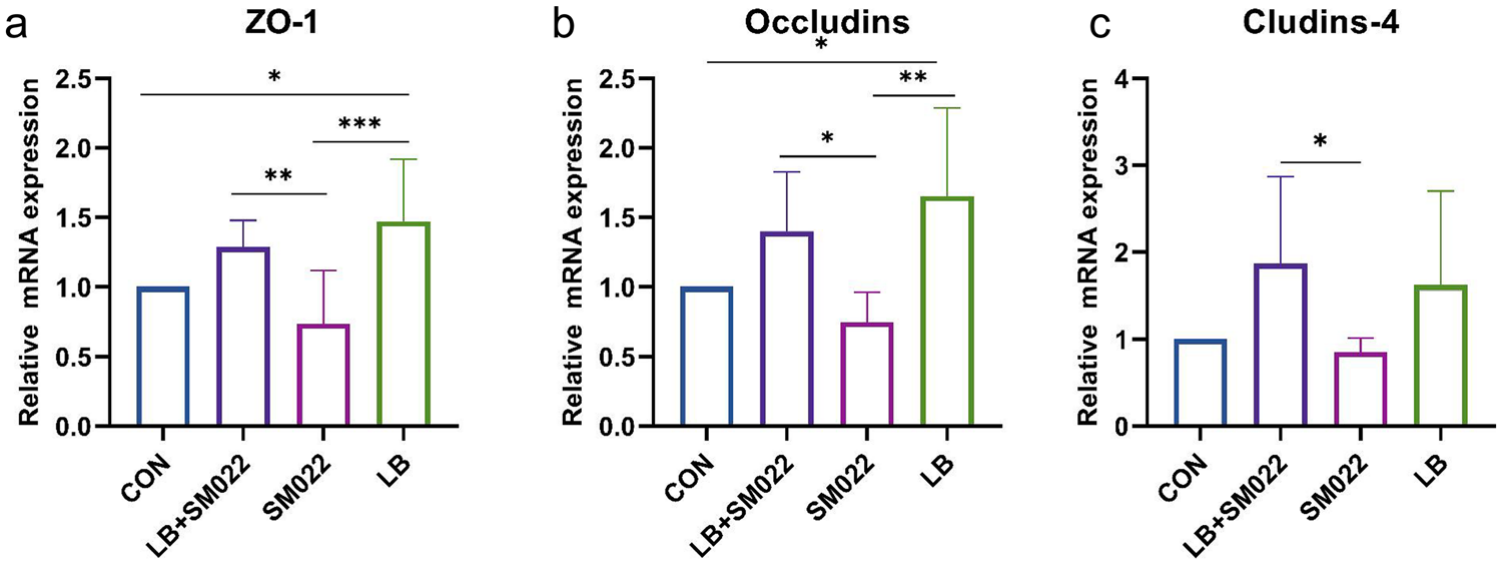
mRNA relative expressions of tight junction proteins (ZO-1, occludins, claudin-4) in the jejunum of mice treated or not during 10 days by *L. buchneri* GX0328-6 and then infected or not with *Salmonella* typhimurium SM022. (*n* = 6) *P < 0.05, **P < 0.01, ***P < 0.001 (one-way ANOVA)

Figure 5a shows that the mRNA expression of the cytokine IL-6 with potential pro-inflammatory function was significantly lower in the jejunum of the prophylactic group compared to the group of positive controls (*P* < 0.005). Meanwhile, there was a trend of decreasing but not yet statistically significant mRNA expression for the anti-inflammatory cytokine IL-10 (Fig. 5b). On the other hand, both groups treated by the prophylactic group and the *L. buchneri* group showed an increase of the Ang4 mRNA expression, while the mRNA expression of REG III decreased. Our data showed that *Salmonella* typhimurium significantly upregulated the expression of REG III mRNA, while it downregulated in the prophylactic group (Fig. 5c, d). In addition, our data represented that *Salmonella* typhimurium decreased the expression of jejunal ZO-1, occludins, and claudins-4, but the mRNA expression of all three was significantly increased (*P* < 0.05) when pretreated the mice with *L. buchneri* GX0328-6 before infection with *Salmonella* typhimurium (Fig. 6).

#### L. buchneri GX0328-6 Could Regulate Intestinal Microflora in Mice

**Fig. 7:**
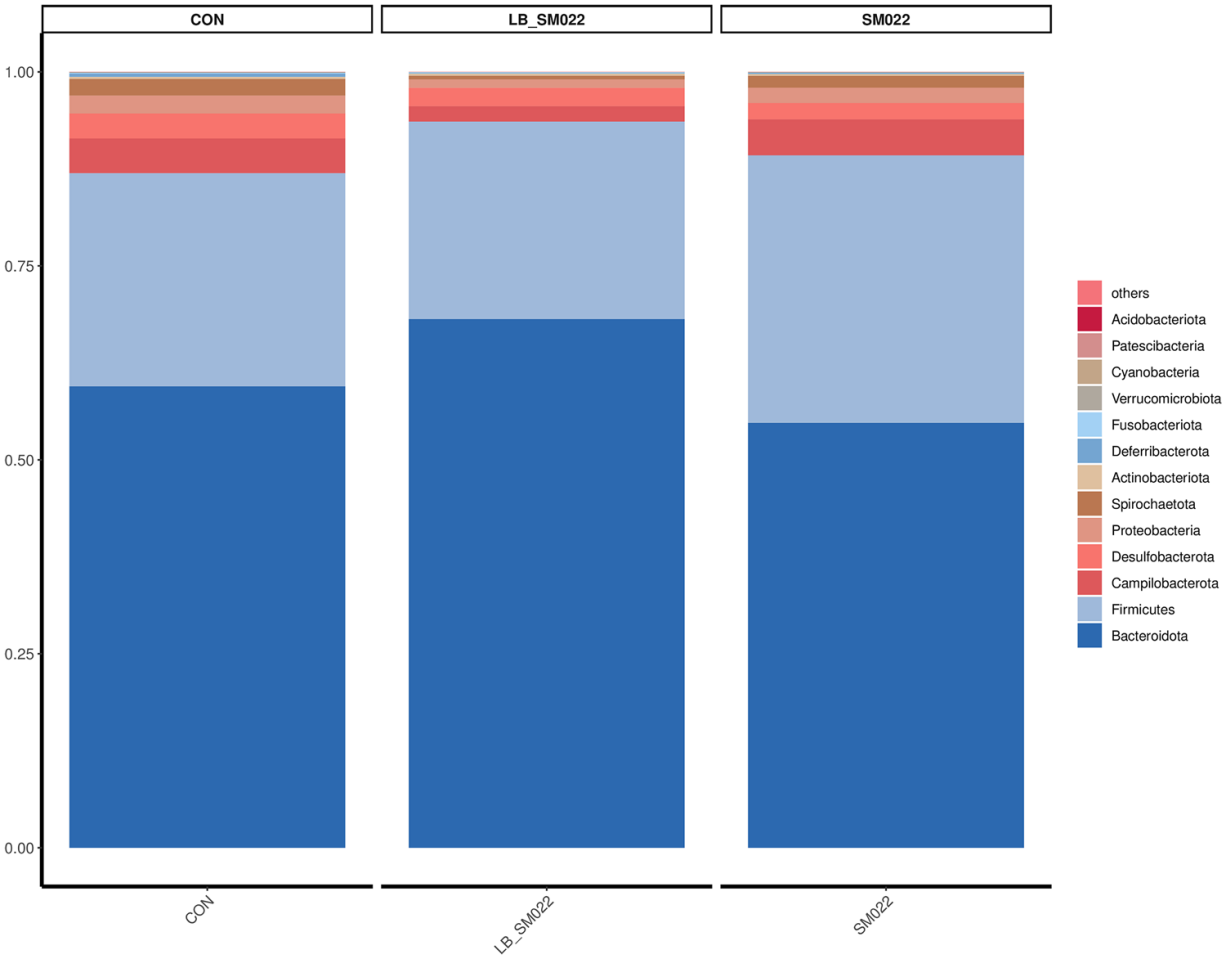
Effect of *L. buchneri* GX0328-6 on cecal microbiota of mice infected with *Salmonella* typhimurium at phyla level (*n* = 3)

**Fig. 8:**
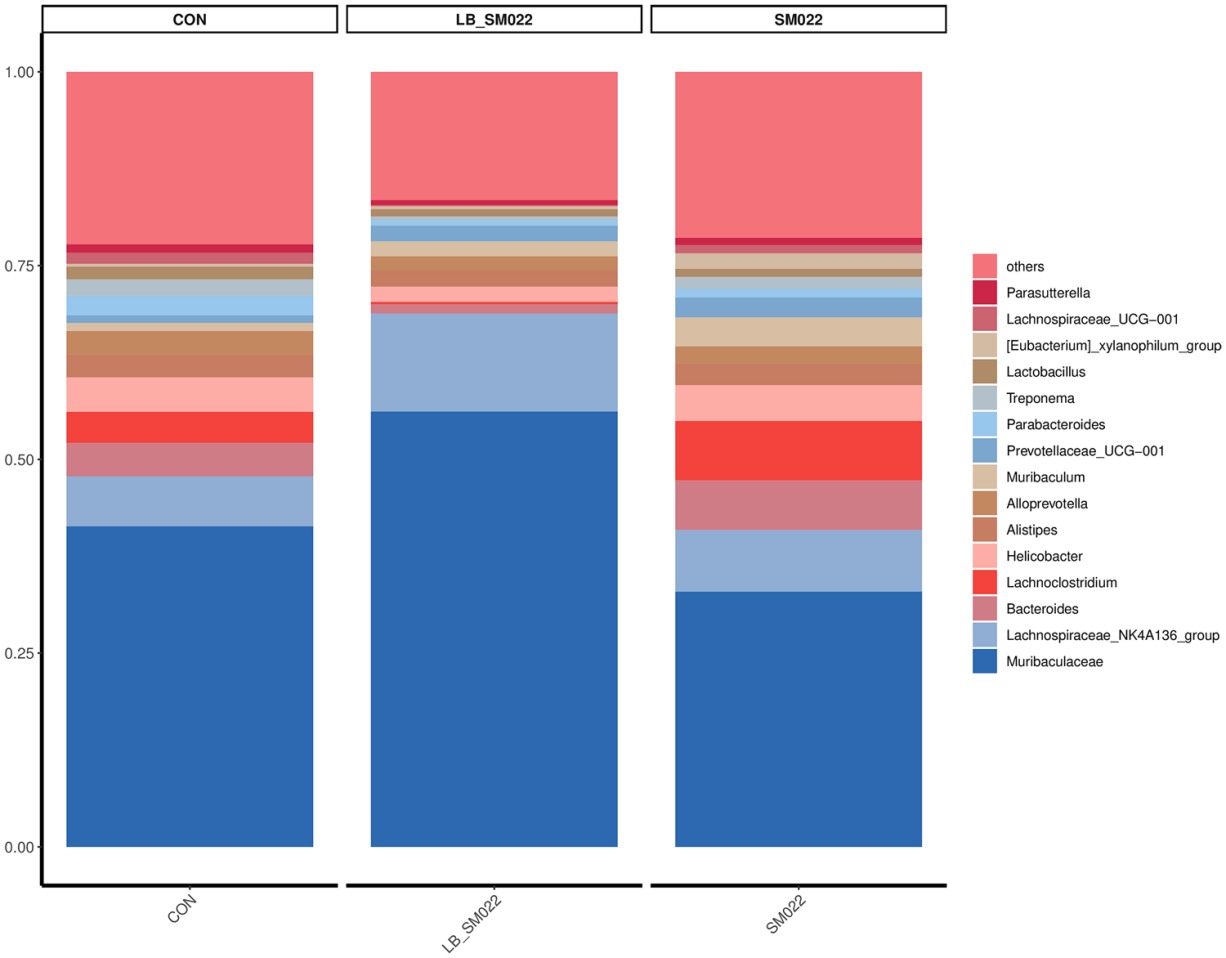
Effect of *L. buchneri* GX0328-6 on cecal microbiota of mice infected with *Salmonella* typhimurium at genus level (*n* = 3)

**Fig. 9:**
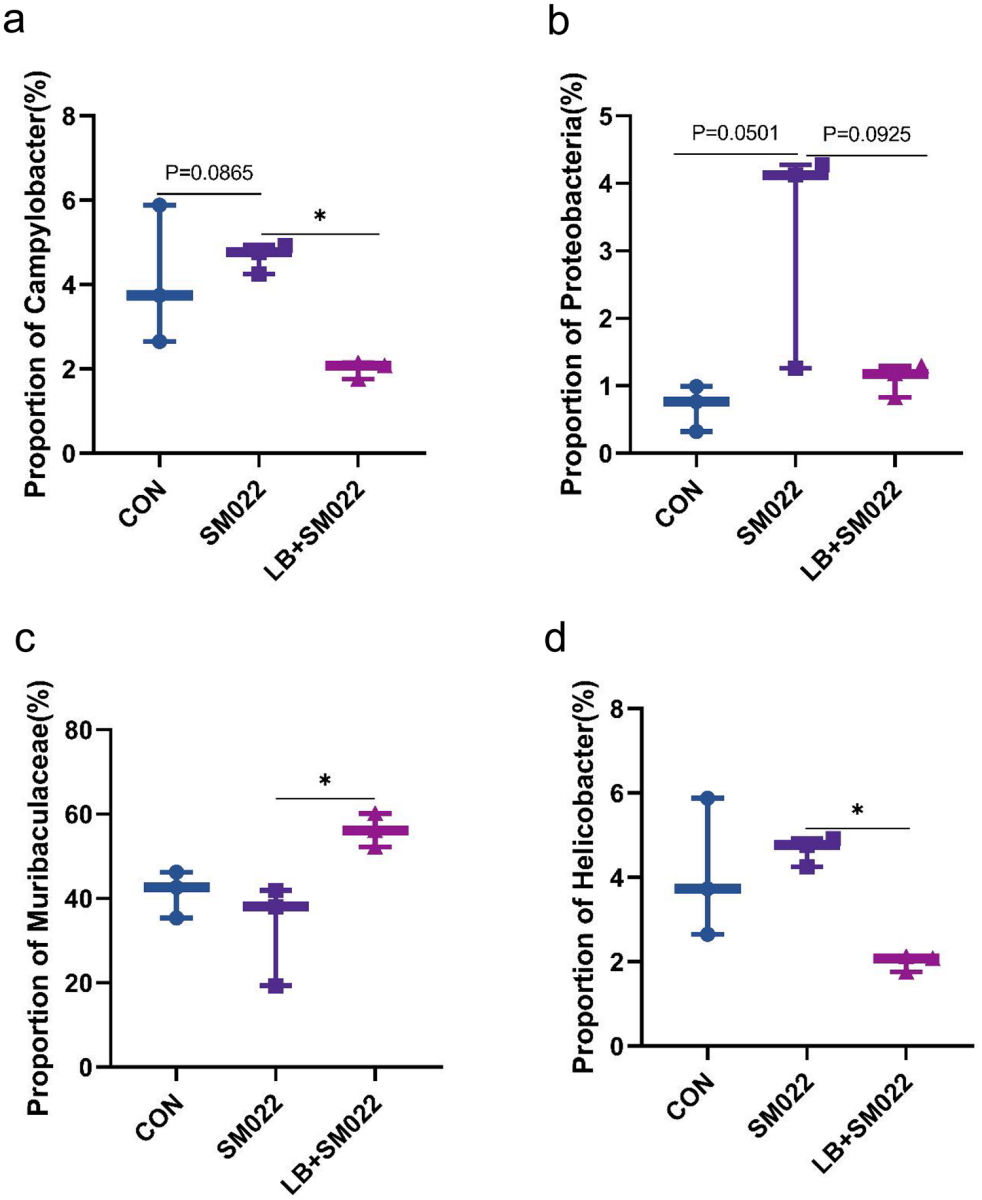
Comparison of relative abundance of significantly different microbial groups at the level of phyla (**a**, **b**) and genus (**c**, **d**). Mann–Whitney *U* test was used for statistical analysis, and the error-free rate (FDR) was corrected. It was compared with DFE + PBS mice (*n* = 3) *P < 0.05 (one-way ANOVA)

Using Illumina MiSeq 16S amplification and sequencing technique, we investigated the effects which *L. buchneri* GX0328-6 makes on modifying the population of cecum microbiota. As shown in Figs. 7 and 8, at the phylum level, compared to the negative control group, the relative proportion of *Bacteroidota* (54.78%) decreased in positive controls, while the relative proportion of their *Firmicutes* (34.48%) increased. Compared to the negative control group, the proportion of *Bacteroidota* (54.78%) in the positive group decreased, Firmicutes (34.48%) increased, and *Campylobacter* (4.66%), *Proteobacteria* (1.98%), and *Spirochaeta* (1.53%) did not show significant changes, while the prevention group not only reduced the increase in *Firmicutes* (25.45%) caused by *Salmonella* typhimurium SM022, but also significantly reduced the proportion of* Campylobacter* (1.99%) (*P *< 0.05) and *Proteobacteria *(1.09%) (*P *= 0.0925) (Fig. 9a, b). The relative proportion of the harmful bacteria, *Spirochaetota* (0.49%), was also reduced, although this change was not statistically significant. In addition, the relative proportion of *Bacteroidota* (68.16%) was also increased. Compared to the negative control group, the *Muribiculaceae* (32.94%) decreased in the positive control group, while the relative proportion of *Muribaculum* (3.75%), *Prevotellaceae_UCG-001* (2.59%), *Lachnocolstridium* (7.64%), and *Ruminococcus* (0.68%) all increased. No significant changes were observed for the *Helicobacter* (4.65%). Additionally, compared with the positive control group, the *L. buchneri*-pretreated group (LB_SM022), an increased population of the *Lachnospiraceae*_NK4A136_group (12.65%) was observed together with a significant increase of the *Muribiculaceae* (56.71%) (*P* < 0.05) (data represented in Fig. 9c). Furthermore, it also inhibited the increasing of *Muribaculum* (1.93%), *Prevotellaceae_UCG-001* (2.04%), *Bacteroides* (1.22%), *Lachnocolstridium* (0.28%), and *Helicobacter* (1.98%) after the infection of *Salmonella* typhimurium SM022. Particularly, it significantly inhibited the population expenditure of *Helicobacter* (1.98%) (*P* < 0.05) (Fig. 9d).

## Materials and Methods

### The Preparation of Bacterial Strains

The testing strain, *Salmonella* typhimurium SM022, is a mutant strain of the wild-type *Salmonella* typhimurium ATCC 14028s [39]. The concentration of 1×108CFU/mL of Salmonella typhimurium SM022 we choose for subsequent experiments depended on our preliminary experiment on the median lethal dose of Salmonella typhimurium SM022. The *Lentilactobacillus buchneri* (*L. buchneri*) was isolated from the traditional Chinese fermented plant food sauerkraut collected in Guangxi and named *Lentilactobacillus buchneri* GXNN20210328-6 (*L. buchneri* GX0328-6). The sequence of *L. buchneri* GX0328-6 was uploaded to NCBI by 16S rRNA comparison with the registration number MZ461959.1. According to the World Gastroenterology Organization Global Guidelines on Probiotics and Prebiotics [40] and the International Scientific Association for Probiotics and Prebiotics (ISAPP) expert team, Binda et al. [41] put forward in the probiotic application guide that the recommended amount of probiotics is 10^8^~10^11^CFU/day, while previous studies have reported that Lactobacillus alleviated *Salmonella* typhimurium infection in mice at a concentration of 10^8^CFU/mL [42, 43], and the concentration of 10^8^ CFU for *L. buchneri* GX0328-6 was selected in the present study. *Salmonella* typhimurium SM022 and *L. buchneri* GX0328-6 were grown to log phase in LB broth and MRS broth (Beijing Landbridge Technology Co., Ltd.), respectively. Bacterial cells were collected by centrifugation at 4000 rpm for 10 min, rinsed twice with saline (NS), and then adjusted the concentration of both bacterial suspensions to 10^8^ CFU/ mL before feeding to mice.

### Animal Research

In this study, eighty-eight 4-week-old C57BL/6 mice were housed at 22–26 °C, 20% humidity with a light/dark cycle for 12 h under standardized conditions. All animals were arbitrary feed of standard diet and distilled water. After 1 week of acclimation, mice were randomly divided into 4 groups (*n* = 22). In the negative control group (CON): each mouse was fed with 0.2 mL of saline alone with the standard diet for 10 days; in the *L. buchneri* group (LB): each mouse was fed with 0.2 mL of 10^8^ CFU/mL of *L. buchneri* GX0328-6 solution for 10 days; in the prophylactic group (LB + SM022): each mouse was fed with 0.2 mL 10^8^ CFU/ mL *L. buchneri* GX0328-6 for 10 days, and then each mouse was treated by 0.2 mL 10^8^ CFU/mL *Salmonella* typhimurium SM022 for 1 day; in the positive group (SM022): each mouse was fed with 0.2 mL saline for 10 days, and then each mouse was treated by 0.2 mL 10^8^ CFU/mL *Salmonella* typhimurium SM022 for 1 day. On the 5th day after the *Salmonella* typhimurium SM022 infection, 10 mice from each group were euthanized, and tissue was collected for further analysis, while other mice were used for testing the survival rate.

### The Measurement of the Immune Organ Index and Survival Rate

Each mouse was weighed and euthanized, and the thymus and spleen were collected/weighed respectively after carefully removing surrounding tissue and fat. The weight of each organ was recorded, and the immune organ index was calculated based on the following formula: immune organ index (%) = immune organ weight/mouse body weight × 100%. The weight of each mouse before and after infection with *Salmonella* typhimurium was observed, and the survival curves were plotted based on the observational data.

### The Determination of Salmonella Translocations

The liver, spleen, and cecum samples from each mouse were isolated, weighed, and homogenized in sterile phosphate–buffered saline (PBS, 1:10, w/v). For each organ, the supernatant of homogenized solution was further diluted into a series of decimal dilutions, incubating at 37 °C for 24 h, and then inoculating on bismuth subsulfite agar medium (Guangdong Huan Kai Microbiology Technology Co., Ltd., Guangdong, China) for *Salmonella*-specific enumeration. The expression threshold of *Salmonella* typhimurium translocation was log_10_ CFU/g of each organ sample.

### The Immunoglobulin and Cytokine Analysis

For serum samples, the level of immunoglobulin IgG, IgM, and IgA were quantified by using a mouse-specific ELISA kit (Jiangsu Jingmei Biotechnology Co., Ltd., Jiangsu, China) according to the manufacturer’s instructions. The OD (optical density) value of each sample was measured by a Thermo Scientific microplate reader at 450 nm wavelength.

### The Relative Expression of Cytokines in Jejunum Samples

A small section (approximately 1 cm) of the jejunum sample was collected and stored at − 80 °C in a 1.5-mL RNAse-free EP tube until further analysis. RNA was extracted according to the RNA pure Tissue&Cell Kit (DNase I) (Jiangsu Kangwei Biotechnology Co., Ltd., Jiangsu, China) procedure. The cDNA of each sample was produced by using the HiFi Script cDNA Synthesis Kit (Kangwei Biotechnology Co., Ltd., Jiangsu, China) according to its manual instructions. Quantitative PCR (qPCR) was performed using QuantiTect SYBR Green PCR Kit (Kangwei Biotechnology Co., Ltd., Jiangsu, China). Primer sequences for each amplification are summarized in Table 1. Amplification reactions were performed by using 10 μl of SYBR green master mix, 50 ng of cDNA, and distilled water to make a total volume of 20 μl. For each gene, the expression level of the control group which feed saline was used as inner control. All results were plotted in a bar graph based on folders of changes for the expression of each target gene. The mean and standard deviation (2^−ΔΔCT^) of the cytokine expression level was calculated following the previously published protocol [43].

**Table 1:** Primers and annealing temperature for relative real-time RT-PCR

Gene/accession number	Primer sequence (5′–3′)	Amplification fragment size/bp	Annealing/℃
IL-6/NM_031168.2	F: CCCCAATTTCCAATGCTCTCC	141	62
	R: CGCACTAGGTTTGCCGAGTA		
IL-10/NM_010548.2	F: AGACCAAGGTGTCTACAAGGC	197	59.6
	R: ACGAGGTTTTCCAAGGAGTTGT		
Ang4/NM_177544.4	F: CTCTGGCTCAGAATGTAAGGTACGA	302	62
	R: GAAATCTTTAAAGGCTCGGTACCC		
REGIII/NM_011260.2	F: CGTGCCTATGGCTCCTATTGCT	120	61.6
	R: TTCAGCGCCACTGAGCACAGAC		
ZO-1/XM_047432991.1	F: TAAACCTGGGGCCATCTCAAC	173	62
	R: CAGAAGGGCTGACGGGTAAAT		
Occludins/NM_001410743.1	F: AAGGTCAAAGAGAACAGAGCAAGAT	98	53.7
	R: GATATTCCCTGATCCAGTCCTCCTC		
Claudins-4/NM_009903.2	F: CCACTCTGTCCACATTGCCT	141	60.5
	R: CCACTCTGTCCACATTGCCT		
β-actin/NM_007393.5	F: GTGACGTTGACATCCGTAAAGA	287	59
	R: GTAACAGTCCGCCTAGAAGCAC		

### The Histological Analysis

For each mouse, the jejunal intestinal segment was collected, briefly rinsed with PBS, and fixed in buffered 4% formaldehyde-PBS solution followed by conventional paraffin embedding. For liver samples, at least two Sects. (4–5 μm/slice) were prepared from each sample, and either hematoxylin or eosin (HE) staining was applied. Histological results were observed by light microscopy (Nikon model eclipse E200 type) and analyzed by a pathologist who was blind to the experimental condition of each sample. To determine villi length and crypt depth, at least 8 villi and crypt foci were measured from different observational fields of each respective tissue section. All images were quantified by using the Image View software (Shanghai Puch Optoelectronics Technology Co., Ltd., Shanghai, China).

### The Analysis of Gut Microbiota

The gut microbiota was observed by using the protocol previously described [44]. Briefly, the V3-V4 region of the 16S rRNA was amplified from the total genomic DNA of cecum contents by using barcoded primers 343F and 798R. PCR amplification products (amplicon) were purified by using Agencourt AMPure XP beads (Beckman Coulter Co., USA) and quantified by the Qubit dsDNA Assay. The 16S rRNA gene amplification products were sequenced and analyzed by Oei Biotech (Shanghai, China). Sequencing data were processed by using the Microbial Ecology 2 (QIIME2) pipeline for quantitative analysis. Sequences were clustered into operational taxonomic units (OTUs) by 97% identity level using the Green genes database (ver. 13_5). Microbial diversity in cecum contents was estimated using alpha diversity including Chao1 index [45] and Shannon index [46] to determine significant differences between groups. Furthermore, in addition, unobserved data were reconstructed based on data from the KEGG pathway database (Genome Net; https://www.genome.jp/kegg/path way.html) to identify functional genes in the microbial communities of the samples [47].

### The Statistical Analysis

The statistical analysis was evaluated by the two-way ANOVA or one-way ANOVA, followed by a post hoc Tukey’s test, with SPSS Statistics version 25.0. The immune organ index of mice was analyzed by two-way ANOVA, and other experimental results were analyzed by one-way ANOVA. The gut microbiota of mice was statistically analyzed using Mann–Whitney *U* Test, with no error detection rate (FDR) correction. Only results with *P* < 0.05 were considered statistically significant.

## Discussion

It has been reported that probiotics could maintain the barrier function of intestinal epithelial cells in mammal model [48–50]. Feeding probiotics inhibited the growth of *Escherichia coli* in the jejunum, colon, and ileum [51]. The *L. buchneri* GX0328-6 strain showed good probiotic properties, which was isolated from sauerkraut, a plant-sourced, traditional fermented food collected from Guangxi province, China. Compared to the positive control group, we observed the gain of average body weight among mice that were pretreated with *L. buchneri* GX0328-6 before being infected by *Salmonella* typhimurium (Fig. 1a). Previous studies have shown that the protection function of probiotic treatment includes the prevention of weight loss under the infection of *Salmonella* among animals [52]. This conclusion is consistent with the results of the present study. Figure 1b shows that feeding the *L. buchneri* GX0328-6 as a pretreatment before the *Salmonella* typhimurium infection could significantly increase the survival rate from 50% (mice without GX0328-6 treatment) to 91.67%. Additionally, the *L. buchneri* GX0328-6 treatment also decreased the symptoms of the *Salmonella* infection as well as a healthier look of their coat and skin appearance (Appendix 1) and significantly reduced thymic index (*P* < 0.05) (Fig. 1c). This suggests that *L. buchneri* GX0328-6 can effectively alleviate clinical symptoms and mortality in mice during *Salmonella* typhimurium infection. Zhang et al. [53] showed that feeding *L. buchneri* in chicks before the *Salmonella* infection could significantly reduce dysentery and improve survival rate, which is consistent with the results in mice after the treatment of *L. buchneri* GX0328-6 in this study. In contrast, Santos et al. found that *Lactobacillus plantarum *286 failed to protect conventional mice from *Salmonella* typhimurium infection, and there was no statistically significant difference among groups, nor was the survival rate changed [54]. This indicates that not all probiotic strains could improve the survival rate of *Salmonella*-infected animals. It also reaffirms the potential of the *L. buchneri* GX0328-6 as a probiotic in protecting mice from the poor outcome of the *Salmonella* typhimurium infection.

Studies have shown that strong antigen-specific cellular and humoral immune responses are associated with defenses against the *Salmonella* typhimurium infection [55]. Oxidative outbreaks of invasive *Salmonella* typhimurium strains were positively correlated with serum levels of antibodies such as immunoglobulins IgG and IgM [56]. Despite the importance of antibodies in the control of *Salmonella* infection [57], little work has been done on analyzing the regulation of serum antibodies after animals were treated by the *L. buchneri* GX0328-6. This ignited our interest in revealing the role of serum antibodies against *Salmonella* typhimurium. Our data showed (Fig. 2a, b, c) that mice infected with the *Salmonella* typhimurium had significantly increased levels of IgG (*P* < 0.05) and no significant differences in levels of IgM and IgA (*P* > 0.05) compared to controls. Other studies have also found elevated IgG antibody levels during 1–4 weeks after infection with *Salmonella* typhimurium inoculation [58]. Interestingly, feeding *L. buchneri* GX0328-6 alone to mice increased immunoglobulin IgG antibody levels, but IgG expression in mice infected with *Salmonella* typhimurium after pretreatment with *L. buchneri* GX0328-6 was not significantly different from that in mice infected with *Salmonella* typhimurium only. It is possible that *L. buchneri*-induced immunoglobulins form an antigen–antibody binding reaction with *Salmonella* typhimurium, thereby slowing the rise in IgG antibody levels. However, the exact mechanism requires further studies before it can be resolved experimentally.

In the present study, the number of *Salmonella* typhimurium detections was significantly lower in all organs of the prophylactic group pretreated with *L. buchneri* GX0328-6 compared to the positive group (Fig. 2e, f, g). After oral administration of *Salmonella* typhimurium, *Salmonella* typhimurium entered the intestine of mice and adhered to intestinal epithelial cells, thereby promoting bacterial colonization in the intestine through its type I hairs [59]. With the migration of phagocytes from the mesenteric lymph nodes, it eventually spreads throughout the body and invades different organs or tissues such as the liver and spleen, causing various clinical symptoms [60]. Invasion of *Salmonella* typhimurium leads to hepatosplenomegaly and eventually organ failure [61]. In the present study, *L. buchneri* GX0328-6 reduced the load of *Salmonella* typhimurium in these organs and alleviated *Salmonella* typhimurium–induced hepatosplenomegaly (Supplementary Figure) Similar results were observed by Acurcio et al. [43] after feeding BALB/c mice with milk fermented by *Lactobacillus paracasei* NCC 2461 (ST11). In addition, H&E staining results showed that *L. buchneri* GX0328-6 pretreatment significantly reduced liver frequency, hepatocyte damage, and inflammatory cells (Fig. 3). These data suggest that pre-feeding of *L. buchneri* GX0328-6 has a positive effect on inhibiting the proliferation and invasion of *Salmonella* typhimurium in the intestinal tract of mice and has a protective effect on organ damage in mice. Studies have shown that the use of certain probiotics can improve the function of the small intestinal barrier by modulating the immune response, thus blocking the translocation of pathogenic bacteria to sterile organs such as the liver [62]. As a result, the survival, proliferation, and spread of pathogens in visceral organs are controlled. The ability of *L. buchneri* GX0328-6 to reduce liver tissue damage may be related to these mechanisms. Interestingly, studies have shown that the spread of *Salmonella* to the liver and spleen is also associated with increased permeability of the intestinal barrier [60]. In contrast, the mechanism of action of *L. buchneri* in regulating intestinal barrier function in animals is not known. Therefore, we next looked at the preventive protective effect of *L. buchneri* by measuring the intestinal mucosal barrier. The intestinal mucosal barrier consists of four parts: physical barrier, immune barrier, chemical barrier, and biological barrier, which can effectively protect against the invasion of foreign pathogenic microorganisms.

Our results showed that *Salmonella* typhimurium significantly decreased villi length and villi length to crypt depth ratio and increased jejunal crypt depth in mice. This is consistent with previous findings [63, 64]. In contrast, *L. buchneri* GX0328-6 pretreatment significantly increased mouse jejunal villus height and the ratio of intestinal villus height to crypt depth and significantly decreased mouse jejunal crypt depth (Fig. 4b, c, d). Prevention by *L. buchneri* GX0328-6 effectively counteracted the effects of *Salmonella* typhimurium SM022 on jejunal villus height and the ratio of intestinal villus height to crypt depth in normal mice. It has been shown that the addition of the probiotic *Enterococcus faecalis* to the diet increased jejunal villus height and ileal villus height [65]. In addition, the addition of a variant o*f Bacillus subtilis* to the diet resulted in the same changes [66]. The same results were obtained in the present study. Also, our pathological histological analysis showed that *L. buchneri* GX0328-6 was effective in protecting the integrity of the small intestinal mucosal tissue and that the intestinal tissues were not damaged (Fig. 4a). In addition, prevention by *L. buchneri* GX0328-6 effectively counteracted the effects of *Salmonella* typhimurium SM022 on jejunal villus height and the ratio of intestinal villus height to crypt depth in normal mice. Taken together, these results suggest that *L. buchneri* GX0328-6 protects the integrity of intestinal epithelial tissue. Furthermore, the length of the villi was significantly and positively correlated with the number of villi epithelial cells, and only mature villi cells had the function of nutrient absorption. Therefore, when the villi are long, there are more mature cells, and the nutrient absorption capacity is strong [67]. The crypt depth reflects the generation rate of intestinal epithelial cells, and a shallower crypt indicates an increase in the maturation rate of intestinal epithelial cells and enhanced secretion function. The ratio of villi length to crypt depth (V/C ratio) was used to reflect the functional status of the small intestine, and when the V/C value increased, it indicated that the villi had an increased ability to absorb nutrients, and vice versa [68, 69]. These results also suggest that *L. buchneri* GX0328-6 enhances intestinal absorption in mice.

In this study, the expression of the tight junction protein gene (ZO-1), occludins, and claudins-4 was upregulated in the intestine of mice in both the *L. buchneri* group and the prophylactic group (Fig. 6a, b, c). The expression of the tight junction proteins ZO-1 and occludins was reported to be reduced after *Salmonella* typhimurium infection [70]. Similar results were obtained from our experiments. Interestingly, we also found that *Salmonella* typhimurium also reduced the expression of claudins-4. Proteins such as ZOs, occludins, and claudins are tight junction structures in the intestine [71]. The intestinal epithelial barrier is the main site of nutrient absorption in the intestine and the first barrier for animals to resist pathogenic microorganisms and protect the organism from invasion by pathogenic microorganisms, and the intestinal tight junctions are the most critical part of the intestinal epithelium [72]. The ability of *L. buchneri* GX0328-6 to significantly increase these genes in this study may be a result of *L. buchneri* GX0328-6 expressing AvrA, as AvrA has been reported to be an effector in stabilizing intestinal tight junctions such as ZO-1 and occludins [73]. Previous studies have reported that certain probiotics significantly maintain intestinal epithelial barrier function in mammals [48–50]. The experimental results of the present study also supported this idea. These results suggest that *L. buchneri* GX0328-6 can protect the mouse intestine from *Salmonella* typhimurium by increasing the length of intestinal villi, decreasing the depth of intestinal crypts, and increasing the tightly connected structures of the intestine as physical barriers.

*Salmonella* typhimurium induces intense intestinal inflammation in several agricultural animal hosts such as cattle, pigs, and poultry [74–77]. During the inflammatory response of the intestine, the intestinal mucosal barrier is damaged, leading to the recruitment of immune cells at the site of inflammation [78]. Some probiotics have been reported to reduce the severity of experimental colitis and improve intestinal inflammation in mice [79]. However, little has been reported so far on the inflammation-attenuating effect of the probiotic *L. buchneri* in vivo. Studies have shown that feeding probiotics significantly increased IL-6 and IL-10 levels in broiler liver [80] and reduced the expression of inflammatory factors in weaned piglets [81], which had a modulating effect on the proliferation and differentiation of immune system cells. While there are relatively few applications and studies on *L. buchneri* in the intestine to regulate IL-6 and other intestinal mucosal immunity, therefore, in this study, we measured IL-6 and IL-10 contents in the jejunum of C57BL/6 mice to investigate the effect of *L. buchneri* GX0328-6 on intestinal mucosal immunity in mice. Our data showed that the gene expression of IL-6 was significantly reduced in the jejunum of mice in both the prophylactic and *L. buchneri* groups compared to the positive group. In contrast, administration of *L. buchneri* GX0328-6 alone significantly increased IL-10 gene expression, although IL-10 was also increased in mice pretreated with *L. buchneri* GX0328-6 and then infected with *Salmonella* typhimurium (Fig. 5a, b). This finding is consistent with the development of probiotics for the treatment of intestinal inflammation [67–69]. IL-6 is commonly considered a pro-inflammatory cytokine produced by classically activated macrophage 1, which has a pro-inflammatory effect on chronic inflammation and autoimmunity [82]. IL-10, on the other hand, is produced by selectively activated macrophage 2 and is usually considered an anti-inflammatory cytokine [83]. IL-10 is closely associated with the prevention of mucosal inflammation by acting on Treg cells or macrophages to prevent inflammatory responses [84]. The results of this study suggest that *L. buchneri* GX0328-6 ameliorates intestinal inflammation by modulating inflammation-associated cytokines and thereby enhancing intestinal immune barrier function.

In addition to the above immune and physical barriers, the intestinal mucosal barrier also has a chemical and microbial barrier. The mucus layer of the chemical barrier is an important line of defense to prevent pathogenic microorganisms from directly contacting intestinal epithelial cells, and in combination with antimicrobial substances secreted into the mucus layer such as antimicrobial peptides (AMPs) including defensins, substances such as lysozyme and endogenous antimicrobial peptide-like substances defensins and regenerating islet derived protein 3 (REGIII), among others, constitute a chemical barrier [85, 86]. Antimicrobial REG III proteins play an important role in maintaining intestinal homeostasis by spatially separating bacteria, preventing potentially harmful immune responses, and protecting the host from infection [87–89]. In addition, it has been shown that Ang4 may have antimicrobial effects [89]. Therefore, we determined the expression of the antimicrobial peptide Ang4 and REG III by qPCR assay to investigate the effect of *L. buchneri* GX0328-6 on the intestinal mucosal barrier in mice infected with *Salmonella* typhimurium. In this study, infection with *Salmonella* typhimurium significantly upregulated the mRNA expression of REG III, while pretreatment with *L. buchneri* GX0328-6 followed by infection with *Salmonella* typhimurium significantly decreased the mRNA expression of REG III and also significantly increased the mRNA expression of Ang4. In addition, administration of *L. buchneri* alone also significantly increased the mRNA expression of Ang4 (Fig. 5c, d). Our results suggest that *L. buchneri* GX0328-6 can promote the intestinal secretion of the antimicrobial substance Ang4 to defend against *Salmonella* typhimurium invasion. These results are consistent with the translocation results we observed for *Salmonella* typhimurium. *L. buchneri* has been shown to significantly induce the expression of REG III at the ileal crypt, ileal villi, and colon after intestinal colonization [90]. However, the results of the present study were contrary to this. Our results found that pretreatment with *L. buchneri* GX0328-6 followed by reinfection significantly downregulated REG III expression; instead, *Salmonella* typhimurium resulted in upregulation of REG III expression. It is possible that REG III, an important repressor in the natural immune system, is abundantly expressed after intestinal damage and enhances natural immune defense in the early stages of inflammation [89]. One study has shown that REG III protein is also consistently increased with increasing levels of inflammation [91]. This also suggests to us that the abnormally high REG III did not suppress the amount of *Salmonella* typhimurium colonization in the intestine of mice well; instead, the intestinal tissues were further disrupted. Therefore, when there is a strong inflammatory response in the mouse intestine, REG III may be regulated by these inflammatory factors and promote abnormal REG III expression with the increase of inflammation. And at this time, the intestinal immune system is severely disrupted only by the massive expression of REG III can no longer inhibit the invading microorganisms; instead, pro-inflammatory cytokines may interact with REG III to play an important role in the amplification of inflammation, but the specific mechanism still needs further study. In addition, *L. buchneri* GX0328-6 pre-treatment followed by reinfection significantly downregulated REG III expression. This suggests that *L. buchneri* GX0328-6 alleviated the intestinal inflammatory pathway likely by affecting inflammation-associated cytokines and thus reducing the abnormal expression of REG III. However, the specific regulatory pathways and mechanisms of *L. buchneri* GX0328-6 on related cytokines and REG III need to be further investigated.

The microbial barrier consists of a large and diverse community of microorganisms located in the intestinal lumen [92]. Currently, gut microbes have become an integral part of human health research [93]. Gut microbes form a symbiotic ecosystem, which maintains the homeostatic balance in humans and animals. However, this balance may be disrupted by pathological conditions that interfere with intestinal physiology [94]. Studies have shown that probiotics not only improve the intestinal tract and promote the reconstruction of the intestinal mucosa [95] but also improve the intestinal microecology by producing different metabolites that inhibit the growth of pathogenic bacteria and promote the growth of beneficial bacteria [96, 97]. For example, strains of *Lactobacillus paracasei* can be used to prevent *Salmonella* typhimurium infections [43]. It has been shown that the diversity and abundance of bacterial species is an aspect of a healthy intestinal microbiota [98]. To investigate whether *L. buchneri* GX0328-6 could maintain the microbial barrier in the intestine by modulating microorganisms, we determined the intestinal flora characteristics of mice in the control, prophylactic, and positive groups. In the present study, *Salmonella* typhimurium infection of C57BL/6 mice was able to reduce the abundance of intestinal *Bacteroidetes*. while *L. buchneri* GX0328-6 not only enhanced the population of *Bacteroidetes* to some extent but also inhibited the colonization of the intestine by certain pathogens (Figs. 7, 9). *Bacteroidetes* is a beneficial bacterium, one of the two most abundant phyla in the intestinal microbiome, that directly mediates the metabolism of carbohydrates, steroids, bile acids, and sugars [99, 100]. Furthermore, our results suggest that *Salmonella* typhimurium infection of C57BL/6 mice altered the structure of the mouse intestinal microbial community by increasing the relative abundance of pathogens of *Protebacterota* and *Campilobacterota*. In contrast, early administration of *L. buchneri* GX0328-6 reduced the proportion of harmful bacteria such as *Protebacterota*, *Campilobacterota*, and *Helicobacter* (Figs. 7, 9). Alterations in intestinal morphology, function, and bacterial flora have been reported to be caused by inflammation. Pathogenic intestinal flora may negatively affect the nutrition of patients and their metabolic efficiency by decreasing microbiota diversity, thus reducing the production of beneficial metabolites [101]. *Salmonella* infection leads to an increase in the number of potential pathogens and parthenogenic anaerobes in the cecum microbiota directly disrupting the intestinal flora ecosystem, which leads to malnutrition of the intestinal flora and causes intestinal inflammation [102, 103]. After feeding *L. buchneri* GX0328-6, the contents of the mouse cecum showed an increase in the dominant flora, such as the *Bacteroidetes*, and a decrease in the harmful flora, such as *Campilobacterota*, with changes in their colony structure, which may be due to the fact that *L. buchneri* GX0328-6 promotes the colonization of beneficial flora, decreases the number of harmful bacteria, and attenuates the inflammatory response caused by harmful bacteria through the production of different metabolites. These results are consistent with the results observed in the immune barrier in which the pro-inflammatory cytokine IL-6 was reduced, and the anti-inflammatory cytokine IL-10 was elevated in the intestinal mucosal tissue. Thus, *L. buchneri* GX0328-6 prophylaxis can effectively reduce the disruption of intestinal flora structure.

Our results found that *Salmonella* typhimurium infection of C57BL/6 mice increased the abundance of *Lachnocolstridium*, *Muribaculum*, *Prevotellaceae*_UCG-001, and *Ruminococcus*. In contrast, feeding *L. buchneri* GX0328-6 reduced the abundance of these flora and *Bacteroides* (Fig. 8). *Lachnocolstridium*, which constitutes an important component of the intestinal flora, can exert anti-inflammatory effects and plays a role in homeostasis in vivo [104]. *Prevotellaceae* has been shown to increase the severity of DSS-induced colitis in mice [105]. And *Prevotella* and *Bacteroides* are the main species in the healthy human intestinal flora that produce acetate and propionate from complex carbohydrates to provide nutrition and maintain the normal physiological functions of the intestine [106]. *Salmonella* typhimurium infection of C57BL/6 mice increased the abundance of *Lachnocolstridium*, *Muribaculum*, *Prevotellaceae*_UCG-001, and *Ruminococcus*. It may be that the organism’s microflora is resistant to foreign invasive pathogens. In addition, compared to the positive group, the prevention group showed an increased abundance of *Muribiculaceae* and *Lachnospiraceae*_NK4A136_group, *Muribiculaceae* formerly known as S24-7 family, which is the main bacterial group in the mouse intestine [107, 108]. The relative abundance of *Muribaculaceae* was negatively correlated with pro-inflammatory cytokines and positively correlated with the expression levels of tight junction protein and mucin 2 [109]. The *Lachnospiraceae*_NK4A136_group is a representative butyrate-producing bacterium that maintains intestinal barrier integrity in mice and is negatively correlated with intestinal permeability. Butyrate, one of the major SCFAs produced by the microbiota, is important in maintaining gastrointestinal health due to its ability to enhance epithelial barrier integrity and inhibit inflammation [110]. Therefore, *Muribiculaceae* and *Lachnospiraceae*_NK4A136_group are important in maintaining the normal state of the intestine in mice [111]. It also indicates that *L. buchneri* GX0328-6 can prevent damage to intestinal tissues by *Salmonella* typhimurium by increasing the abundance of beneficial bacteria in the intestinal flora and by inhibiting certain pathogenic bacteria.

## Conclusion

This study showed that the *L. buchneri* GX0328-6 attenuated the symptoms caused by the *Salmonella* typhimurium-infection among C57BL/6 mice, significantly increased their survival rate, and had a significant preventive protection effect. The possible mechanism is through the ability of the *L. buchneri* GX0328-6 to modulate the level of serum immunoglobulins, enhance the intestinal mucosal barrier, and reduce the effect of the *Salmonella* typhimurium on the intestinal invasion of mice. Immunoglobulin levels did not change in mice after infection with *Salmonella* typhimurium probably because *L. buchneri*-induced immunoglobulins formed an antigen–antibody binding reaction with the *Salmonella* typhimurium during the infection cycle. Nevertheless, pre-treated with *L. buchneri* GX0328-6 followed by the *Salmonella* typhimurium infection also modulated the intrinsic immunity of mice. The *L. buchneri* enhanced the mucosal barrier and absorptive capacity of mouse jejunum by upregulating the expression levels of tight junctions such as ZO-1, occludins, and claudins-4 and increasing the ratio of villi length and villi length to crypt depth in mouse jejunum and decreasing the crypt depth through GX0328-6. Interestingly, the *L. buchneri* GX0328-6 reduced intestinal proliferation and invasion of *Salmonella* typhimurium by promoting the expression of antimicrobial peptides in the mouse intestine, and reduced intestinal inflammation and systemic spread in mice by downregulating the expression of pro-inflammatory cytokine IL-6 and promoting the expression of anti-inflammatory cytokine IL-10 in the jejunum. In addition, the *L. buchneri* GX0328-6 increased the relative abundance of beneficial bacteria and decreased the relative abundance of harmful bacteria in the cecum microflora by modulating the microflora in the cecum contents.

## Electronic supplementary material

Below is the link to the electronic supplementary material.

Supplementary file1 Appendix 1 The pathological changes of liver(a) and spleen(b) and the clinical symptoms of mice. (ZIP 55699 KB)

## Data Availability

The data and materials presented in this study are available on request from the corresponding author.
